# Health-related quality of life in patients with myalgic encephalomyelitis/chronic fatigue syndrome: an Australian cross-sectional study

**DOI:** 10.1007/s11136-019-02411-6

**Published:** 2020-01-22

**Authors:** N. Eaton-Fitch, S. C. Johnston, P. Zalewski, D. Staines, S. Marshall-Gradisnik

**Affiliations:** 1 School of Medical Science, Griffith University, Gold Coast, Australia; 2 National Centre for Neuroimmunology and Emerging Diseases, Menzies Health Institute Queensland, Griffith University, Gold Coast, Australia; 3 Consortium Health International for Myalgic Encephalomyelitis, Griffith University, Gold Coast, Australia; 4 Department of Hygiene, Epidemiology and Ergonomy, Uniwersytet Mikolaja Kopernika Collegium Medicum, Bydgoszcz, Poland

**Keywords:** Myalgic encephalomyelitis, Chronic fatigue syndrome, Health-related quality of life, SF-36

## Abstract

**Background:**

Myalgic encephalomyelitis/chronic fatigue syndrome (ME/CFS) is a serious and debilitating disorder associated with significant disruptions in daily life including. This study aimed to examine the impact of sociodemographic and patient symptom characteristics on health-related quality of life (HRQoL) of Australians with ME/CFS.

**Methods:**

Self-reported data collected from 480 individuals diagnosed with ME/CFS were obtained between August 2014 and August 2018. This cross-sectional survey analysed sociodemographic, symptom characteristics and HRQoL according to the 36-Item Health Survey (SF-36). Multivariate linear regression models were used to determine ME/CFS symptoms associated with eight domains of HRQoL.

**Results:**

Reported HRQoL was significantly impaired in ME/CFS patients across all domains compared with the general population. Scores were the lowest for physical role (4.11 ± 15.07) and energy/fatigue (13.54 ± 13.94). Associations with females, higher body mass index (BMI), employment status, cognitive difficulties, sensory disturbances and cardiovascular symptoms were observed in the physical functioning domain. Impaired pain domain scores were associated with high BMI, annual visits to their general practitioner, flu-like symptoms and fluctuations in body temperature. Reduced well-being scores were associated with smoking status, psychiatric comorbidity, cognitive difficulties, sleep disturbances and gastrointestinal difficulties.

**Conclusion:**

This study provides evidence that ME/CFS has a profound and negative impact on HRQoL in an Australian cohort.

## Introduction

Myalgic encephalomyelitis (ME), also referred to as chronic fatigue syndrome (CFS), is a serious condition clinically defined by dysregulation of the central nervous, cardiovascular and immune systems, endocrine dysfunction, and impaired cellular energy metabolism and ion transport [1, 2]. Due to the ambiguous nature of ME/CFS, there is no single diagnostic test; however, diagnosis relies on the fulfilment of criteria after unsuccessful differential diagnosis. In 1994, the Centers for Disease Control and Prevention (CDC) published the Fukuda Criteria to evaluate and classify ME/CFS patients and provide a basis for diagnosis [3]. A case of ME/CFS is defined under these criteria by the presence of unexplainable chronic fatigue that is not alleviated by rest. Furthermore, at least four additional concurrent symptoms including sore throat, tender lymph nodes, muscle and/or joint pain, impaired cognition and sleep disturbances are necessary for diagnosis. Revised protocols birthed the Canadian Consensus Criteria (CCC) (2003) and the International Consensus Criteria (ICC) (2011) [1, 4]. These revised definitions introduced the following symptoms post-exertional neuroimmune exhaustion accompanied by numerous neurological, cardiovascular, autonomic and neuroendocrine manifestations.

The prevalence of ME/CFS is difficult to determine due to the absence of a diagnostic test; however, estimates believe that 200,000 Australians have been diagnosed or report symptoms of ME/CFS [5]. Epidemiological studies have commented on the severity spectrum of ME/CFS. Patient severity is defined by a significant reduction in the patient’s premorbid activity level. Mild patients self-report an approximately 50% reduction in pre-illness activity level including ability to maintain employment and social interactions. Approximately 25% of ME/CFS patients are considered severe, being primarily bed-bound, while moderate patients are bound to their home [6]. Relatively few patients with ME/CFS completely recover [7], with a recovery rate below six% and increased disability in 10–20% of patients over time [8].

The Medical Outcomes Study Short-Form General Health Survey (SF-36) is widely used to measure self-reported health-related quality of life (HRQoL) [9, 10]. Previous qualitative investigations have used SF-36 to report distinctive patterns of impairment in ME/CFS patients and to distinguish these patients from other conditions. The SF-36 consists of domains separately referred to as physical functioning, physical role, body pain, general health, social functional, emotional health and fatigue/energy. A previous investigation by Reeves et al*.* defined SF-36 scores in mild, intermediate and severe ME/CFS patients [11]. According to this empiric ME/CFS case definition, the disability criterion is met by scoring the 25th percentile on any one of the following four SF-36 scales: Physical Functioning (≤ 70), Physical Role (≤ 50), Social Functioning (≤ 75) or Emotional Role (≤ 66.7) [11].

HRQoL is an important assessment in chronic conditions to monitor disability as a predictor of health service requirements [12]. The aim of this study was to further understand contributions of ME/CFS pathophysiology associated with reduced HRQoL and identify aspects of health that are most limited. We hypothesise that particular sociodemographic and symptom characteristics would be associated with significantly reduced HRQoL. Identifying these symptoms may improve patient management and health resource allocation.

## Method

### Study design and setting

This study utilises a cross-sectional survey during a four-year period from August 2014 to August 2018 that collected patient level data for biological investigations into ME/CFS at NCNED (National Centre for Neuroimmunology and Emerging Diseases) research centre. ME/CFS patients across Australia volunteered in this survey in response to online recruitment advertisements. To be eligible for this study, participants were required to: (1) report experiencing ME/CFS illness; (2) be between 18 and 65 years of age; and (3) be a resident of Australia. Informed consent was obtained by agreeing to terms and conditions disclosed online or signing a hard copy sent via email. This study was approved by Griffith University Human Research Ethics Committee (HREC reference: MSC0413).

### Data collection

Participants completed a survey through an online application (LimeSurvey, Carsten, Schmitz, Hamburg, Germany) or by hard copy in the mail. Data from completed hard copy questionnaires were returned to NCNED and manually entered into LimeSurvey by a member of the research team. Items in the survey were developed by the authors and items included sociodemographic details, a 60-item checklist on fatigue and ME/CFS symptoms repurposed from the Fukuda [3], CCC [4] and ICC [1] diagnostic definitions. Participants were asked to disclose other illnesses as diagnosed by a physician and medications routinely administered within two weeks of completing the questionnaire. All participants were anonymised using an alpha-numeric code.

### Sociodemographic characteristics

Sociodemographic data were collected. Age was measured at time of response and gender (male or female). Body Mass Index (BMI, kg/m^2^) was calculated by participants according to self-reported weight (kg) and height (cm). BMI was used to categorised participants as underweight (< 18.5), normal (18.5–24.9), overweight (25.0–29.9) and obese (> 30.0). Participants reported their highest level of education obtained and assessed according to primary school, high school, professional training, undergraduate degree or postgraduate degree. Participants reported their working status and was analysed as full time, part time, disability pension, retired or unemployed. Smoking status was reported as either smoker or non-smoker.

Psychological comorbidity was assessed according to whether a participant reported concurrent diagnosis of a psychological condition by a physician (e.g. depression and anxiety ). Participants reported the number of visits to a general practitioner per. Participants were also asked whether they identified with an infectious event such as cold, flu, bacteria or viral infection prior to the onset of their illness and examined according to ‘yes’ or ‘no’ responses. Age of onset, defined as age the participant noticed ME/CFS symptoms had caused a disruption to daily or vocational activities.

### Symptom characteristics

To coincide with the study measures of HRQoL, this investigation asked participants to also report the impact of seven symptom categories during the past four weeks. These categories included the following: (i) cognitive difficulties (slowed thought, impaired concentration and short term memory loss); (ii) pain (headaches, muscle pain and multi-joint pain); (iii) sleep disturbances (insomnia, prolonged sleep, reversed sleep cycle); (iv) cardiovascular symptoms (orthostatic intolerance, heart palpitations, light headedness and dizziness); (v) respiratory symptoms (air hunger, laboured breathing); (vi) body temperature intolerances (subnormal body temperature, abnormal sweating episodes, hot flushes and cold extremities); and (vii) sensory, perceptual and motor disturbances (sensitivity to touch, light, odour, taste, sound, movement, and poor balance or coordination). Symptom severity was measured by four-point scale corresponding to non, mild, moderate to severe and was analysed dichotomously as non-mild vs. moderate–severe.

Participants reported symptoms and illnesses were reviewed by the authors of this manuscript to exclude any potential major illnesses reported as active, recurrent or not completely resolved, as this may be an alternative explanation for symptoms. Other diagnoses leading to exclusion include, but not limited to the following: thyroid-related diseases, high BMI, diabetes, insomnia, cardiovascular disease, autoimmune diseases or malignancies. To be included in this study, participants were required to fulfil the Fukuda case definition [3]. To fulfil this criteria fatigue was required to be present for at least six months leading to a significant disruption to daily and vocational activities. Additionally, this fatigue must have been accompanied by at least four of the following symptoms: (i) post-exertional malaise; (ii) cognitive difficulties; (iii) headache; (iv) sore throat; (v) muscle pain; (vi) multi-joint pain; (vii) unrefreshing sleep and (viii) tender lymph nodes. Accompanying symptoms should not have preceded the onset of fatigue and also be persistent or recurring for at least 6 months.

### Health-related quality of life

HRQoL was measured according to the SF-36 Item Health Survey version 1.0 [12]. The survey contains 36 items to measure physical health and mental health of participants during a four-week period. The SF-36 survey is divided into eight domains including: (i) physical functioning; (ii) role limitations due to physical health; (iii) role limitations due to pain; (iv) role limitations due to general health; (v) role limitations due to emotional health; (vi) role limitations due to vitality; (vii) role limitations due to emotional well-being and (viii) role limitations due to social functioning. Each item was assessed by Likert scales, with each response assigned a value ranging from 0 to 100. Scores from each domain were averaged to provide a final score with lower scores indicating reduced HRQoL and high scores indicating better HRQoL. General population SF-36 scores were obtained from the Australian Bureau of Statistics 1995 National Health Survey for comparison with ME/CFS patients [13]. The authors wish to note that more recent data specific to SF-36 general population scores are not available for this comparison.

### Statistical analysis

Statistical analysis was performed to examine ME/CFS symptoms associated with each HRQoL domain from the following categories: (i) physical functioning; (ii) role limitations due to physical health; (iii) role limitations due to pain; (iv) role limitations due to general health; (v) role limitations due to emotional health; (vi) role limitations due to energy/fatigue; (vii) role limitations due to emotional well-being and (viii) role limitations due to social functioning. Multivariate analysis was performed (multi linear regression) using the forward stepwise procedure to determine our final models for each HRQoL domain. Age, sex (male vs. female) and BMI (underweight < 18.5; normal 18.5–24.9; overweight 25.0–29.9; and obese > 30.0) were adjusted in each model prior to analysis and included in analysis irrespective of significance. The Akaike information criteria (AIC) were used to compare regression models and the model with the smallest AIC value was used. Thus, all other health variables were included as variables if *p* < 0.1. We verified basic assumptions for multiple linear regression including normality of residuals and report standardised beta (*β*) coefficients, with corresponding *t* and *p* values. Robustness checks were performed using ordinal logistic regression and due to the non-normal nature of the data, quantile regression was also performed. Any discrepancies in results obtained from these models were recorded in the results. All data presented are mean ± standard deviation (SD) unless otherwise stated. The analysis was conducted using SPSS v.24 software (IBM Corp, USA) and STATA v.14 (StataCorp, Texas, USA).

## Results

### Patient demographics

During the study period of August 2014 to August 2018, 480 ME/CFS patients successfully completed and submitted the questionnaire. All patients reported symptoms fulfilling the Fukuda criteria and no other fatigue related illnesses that may account for their symptoms. Table 1 includes all demographic data of Australian ME/CFS patients who completed the questionnaire. The sample was predominantly female (77.5%), within normal BMI (18.5–24.9) range (45.5%), received the disability pension (28.8%), completed an undergraduate degree (31.3%) and resided in Queensland (44%). Majority of participants identified themselves as non-smokers (90.8%). Approximately half the sample reported experiencing the onset of their symptoms prior to age 30 (49.0%) in addition to reporting undue stress (52.5%) and an infectious event prior to onset (48.3%).

**Table 1:** ME/CFS Descriptive characteristics (*n* = 480)

Age (years, mean ± SD)		46.0 ± 12.3
Gender (*n*, %)		
Female	372	77.5%
Male	108	22.5%
BMI (*n*, %)		
Underweight < 18.5	18	3.8%
Normal 18.5–24.9	218	45.4%
Overweight 25.0–29.9	128	26.7%
Obese > 30	116	24.2%
Work status (*n*, %)		
Full time	88	18.3%
Part time	105	21.9%
Retired	15	3.1%
Unemployed	134	27.9%
Disability pension	138	28.8%
Education (*n*, %)		
Primary school	3	0.6%
High school	108	22.5%
Professional training	110	22.9%
Undergraduate	150	31.3%
Postgraduate	109	22.7%
Smoking status (*n*, %)		
Smoking	44	9.2%
Non-smoking	436	90.8%
Location (*n*, %)		
Queensland	211	44%
New South Wales	103	21.5%
Victoria	71	14.9%
ACT	8	1.8%
Northern Territory	2	0.4%
South Australia	24	5.0%
Western Australia	38	7.9%
Tasmania	9	1.9%
Psychological comorbidity (*n*, %)		
Yes	77	16.1%
No	400	83.9%
WHODAS		
Communication and understanding		35.58 ± 18.13
Mobility		43.32 ± 22.43
Self-Care		17.53 ± 19.73
Getting along		32.74 ± 22.98
Life activities		56.64 ± 27.43
Participation in work/school		58.40 ± 27.26
Participation in society		48.61 ± 22.43
GP visits per year (mean ± SD)		12.0 ± 16.2
Onset age (mean ± SD)		29.9 ± 12.1
Infection onset (*n*, %)		
Yes	232	48.3%
No	248	51.7%
Undue stress onset (*n*, %)		
Yes	252	52.5
No	228	47.5
Severe physical trauma onset (*n*, %)		
Yes	54	11.3
No	426	88.8
Infection with travelling onset (*n*, %)		
Yes	62	12.9
No	418	87.1

*ME/CFS* Myalgic encephalomyelitis/chronic fatigue syndrome, *SD* standard deviation, *BMI* body mass index, *ACT* Australian Capital Territory, *GP* general practitioner

Table 2 includes all ME/CFS symptom descriptive data and associated severity scores. During the 30 days prior to completing the survey, many participants reported experiencing moderate to severe cognitive difficulties (75%), pain (72.3%), and sleep disturbances (73.3%). Approximately half reported experiencing moderate to severe sensory disturbances (56.3%), flu-like symptoms such as sore throat, tender lymph nodes, and sinus issues (53.8%), gastrointestinal disturbances (55.2%) and problems with body temperature (51.7%). A proportion reported experiencing moderate to severe cardiovascular symptoms (19.2%) and breathing difficulties (30.0%).

**Table 2:** ME/CFS symptom characteristics

ME/CFS Symptoms	*n*	(%)
Cognitive difficulties (*n*, %)		
None-mild	120	25.0
Moderate–severe	360	75.0
Pain (*n*, %)		
None-mild	133	27.7
Moderate–severe	347	72.3
Sleep disturbances (*n*, %)		
None-mild	128	26.7
Moderate–severe	352	73.3
Sensory disturbances (*n*, %)		
None-mild	210	43.8
Moderate–severe	270	56.3
Flu-like symptoms (*n*, %)		
Non-mild	222	46.3
Moderate–severe	258	53.8
Gastrointestinal disturbances (*n*, %)		
None-mild	215	44.8
Moderate–severe	265	55.2
Cardiovascular symptoms (*n*, %)		
None-mild	388	80.8
Moderate–severe	92	19.2
Breathing difficulties (*n*, %)		
None-mild	336	70.0
Moderate–severe	144	30.0
Thermostatic instability (*n*, %)		
None-mild	232	48.3
Moderate–severe	248	51.7

*ME/CFS* Myalgic encephalomyelitis/chronic fatigue syndrome

### HRQoL scores in ME/CFS patients compared with general population

General population scores were obtained from the Australian Bureau of Statistics 1995 National Health Survey [13]. This survey presents an overview of health status (e.g. prevalence of disease) and factors which may influence health (e.g. smoking, exercise). The majority of Australians considered themselves as being in good health with 83% reporting their health status as good, very good or excellent. Three-quarters of the population experience one or more long-term condition lasting six months or longer. Common illnesses reported in the general population include asthma (11%), headache (13%), hypertension (10%), short sighted (21%) and arthritis (15%).

The SF-36 health outcomes survey was used to assess HRQoL in ME/CFS patients compared with general population norms for Australians. As reported in Table 3, mean HRQoL scores were significantly reduced in ME/CFS patients across all domains (*p* < 0.001). Scores were particularly low for limitations due to physical health (4.11 ± 15.1) and energy/fatigue (13.54 ± 13.94).

**Table 3:** HRQoL scores of ME/CFS compared to general population (mean ± SD)

	ME/CFS (*n* = 480)	General population (*n* = 15,938)^a^		
			*t*	*p*
Physical functioning	37.7 ± 24.1	82.6 ± 23.9	40.54	< 0.001
Physical role	4.1 ± 15.1	79.9 ± 35.1	47.17	< 0.001
Pain	47.5 ± 25.3	76.8 ± 25.0	25.29	< 0.001
General health	23.9 ± 14.6	71.6 ± 20.3	51.08	< 0.001
Social functioning	24.5 ± 23.5	85.0 ± 22.5	57.97	< 0.001
Energy/fatigue	13.5 ± 13.9	64.5 ± 19.8	56.02	< 0.001
Emotional role	51.9 ± 44.9	82.9 ± 32.3	20.44	< 0.001
Emotional well-being	60.9 ± 18.9	75.9 ± 17.0	18.98	< 0.001

*ME/CFS* Myalgic encephalomyelitis/chronic fatigue syndrome, *SD* standard deviation

^a^Australian National Health Survey

### Symptom and health characteristic HRQoL scores for ME/CFS patients

Multivariate analyses for HRQoL domains including physical functioning, physical role, pain and general health are presented in Table 4. Significantly reduced scores for physical functioning were associated with individuals that were female, had a higher BMI, employment status and cognitive difficulties, sensory disturbances and cardiovascular symptoms. These factors explained 23.6% of total variance (adjusted *R*^2^ = 0.236, *p* < 0.001). Significantly reduced scores for role limitations due to physical health were associated with annual visits to the GP, severe pain symptoms, cognitive disturbances and gastrointestinal disturbances (adjusted *R*^2^ = 0.075, *p* < 0.001). Significantly reduced scores for pain were associated with higher BMI, employment status, annual visits to the GP, pain symptoms, flu-like or immunological symptoms, and body temperature complaints (adjusted *R*^2^ = 0.497, *p* < 0.001). The factors associated with significantly reduced general health scores were associated with higher BMI, psychological comorbidities, smoking status, sensory disturbances and flu-like symptoms (adjusted *R*^2^ = 0.086, *p* < 0.001).

**Table 4:** Multivariate analysis of HRQoL physical functioning, physical role, pain and general health domains

	Physical functioning			Physical role			Pain			General health		
	*β*	*T*	*P*	*β*	*t*	*p*	*β*	*t*	*p*	*β*	*t*	*p*
Age	− 0.044	− 1.076	0.283	− 0.086	− 1.908	0.057	− 0.03	− 0.896	0.371	0.071	1.56	0.119
Gender	− 0.156	− 3.809	< 0.001	− 0.007	− 0.155	0.877	0.002	0.07	0.945	− 0.05	− 1.128	0.26
BMI	− 0.100	− 2.434	0.015	− 0.009	− 0.197	0.844	− 0.089	− 2.652	0.008	− 0.087	− 1.885	0.06
Work status	− 0.190	− 4.615	< 0.001				− 0.058	− 1.759	0.079			
Education												
Psychological comorbidity										− 0.077	− 1.684	0.093
Smoking status										− 0.088	− 1.947	0.052
Infectious onset												
GP visits per year				− 0.074	− 1.655	0.099	− 0.071	− 2.117	0.035			
Onset age												
Cognition	− 0.106	− 2.455	0.014	− 0.23	− 5.142	< 0.001						
Pain				− 0.078	− 1.701	0.09	− 0.639	− 18.711	< 0.001			
Sleep												
Sensory	− 0.228	− 5.0273	< 0.001							− 0.114	− 2.489	0.013
Flu							− 0.072	− 2.095	0.037	− 0.181	− 3.944	< 0.001
Gastrointestinal				− 0.093	− 2.061	0.04						
Cardiovascular	− 0.193	− 4.720	< 0.001									
Respiratory												
Temperature							− 0.072	− 2.117	0.035			
*R*^2^			0.247			0.099			0.505			0.099
Adjusted *R*^2^			0.236			0.085			0.497			0.086
*p* value			< 0.001			< 0.001			< 0.001			< 0.001

*HRQoL* health-related quality of life, *BMI* body mass index, *GP* general practitioner

Table 5 shows the results of multivariate analyses for HRQoL domains including social functioning, energy/fatigue, emotional role and well-being. Significantly reduced scores for social functioning were associated with employment status, psychological comorbidities, cognitive difficulties, sensory disturbances and cardiovascular symptoms (adjusted *R*^2^ = 0.13, *p* < 0.001). Significantly reduced scores for energy/fatigue were associated with higher BMI, smoking status, cognitive difficulties and sleep disturbances (adjusted *R*^2^ = 0.084, *p* < 0.001). The factors associated with reduced scores for role limitations due to emotional health were psychological comorbidity, annual GP visits, cognitive difficulties and body temperature complaints (adjusted *R*^2^ = 0.045, *p* < 0.001). Significantly reduced scores for general well-being were associated with higher age, psychological comorbidity, smoking status, cognitive difficulties, sleep disturbances and gastrointestinal disturbances (*R*^2^ = 0.155, *p* < 0.001).

**Table 5:** Multivariate analysis of risk factors and HRQoL social functioning, energy, emotional role and well-being domains

	Social functioning			Energy/fatigue			Emotional role			Well-being		
	*β*	*t*	*p*	*β*	*t*	*p*	*β*	*t*	*p*	*β*	t	*p*
Age	0.032	0.716	0.475	0.078	1.742	0.082	0.018	0.388	0.698	0.131	3.025	0.003
Gender	0.058	1.336	0.182	− 0.082	− 1.873	0.062	0.027	0.608	0.544	0.066	1.544	0.123
BMI	0.049	1.081	0.28	0.096	2.123	0.034	0.005	0.108	0.914	0.064	1.435	0.152
Work status	− 0.104	− 2.349	0.019									
Education												
Psychological comorbidity	− 0.094	− 2.103	0.036				− 0.16	− 3.467	0.001	− 0.234	− 5.283	< 0.001
Smoking status				− 0.104	− 2.336	0.02				− 0.108	− 2.492	0.013
Infectious onset												
GP visits per year							− 0.091	1.99	0.047			
Onset age												
Cognition	− 0.196	− 4.248	< 0.001	− 0.195	− 4.395	< 0.001	− 0.077	− 1.673	0.095	− 0.074	− 1.739	0.083
Pain												
Sleep				− 0.149	− 3.357	0.001				− 0.195	− 4.443	< 0.001
Sensory	− 0.139	− 2.992	0.003									
Flu												
Gastrointestinal										− 0.092	− 2.085	0.038
Cardiovascular	− 0.196	− 4.248	< 0.001									
Breathing							− 0.09	− 1.967	0.05			
Temperature												
*R*^2^			0.147			0.096			0.059			0.169
Adjusted *R*^2^			0.13			0.084			0.045			0.155
*p* value			< 0.001			< 0.001			< 0.001			< 0.001

*HRQoL* health-related quality of life, *BMI* body mass index, *GP* general practitioner

### Robustness check

Due to the nature of some survey responses and the non-normal nature of the data, robustness checks were performed. Difference in statistical significance were reporting for the following using quantile regression: (i) a loss of significance for cognitive difficulties was reported for physical functioning; (ii) for pain a statistical significance was lost for BMI, employment status, annual visits to the GP and temperature instability; (iii) for general health statistical significance was lost for age; (iv) statistical significance was lost for employment status for social functioning; (v) for energy/fatigue a loss of significance was reported for gender, age and smoking status and (vi) for role emotional statistical significance was lost for annual visits to the GP. Ordinal logistic regression was also performed and differences in statistical significance were reported for the following: (i) for physical role statistical significance was lost for age and (ii) for pain statistical significance was lost for employment status.

## Discussion

The present findings represent the largest and most recent investigation of an Australian population for physical and mental health status reported by those experiencing ME/CFS symptoms. This current manuscript for the first time reports important factors to consider in improving patient outcomes and overall HRQoL. Consistent with other investigations, HRQoL outcomes were significantly reduced in ME/CFS patients compared with general population norms. A previous Australian pilot study completed at NCNED compared HRQoL between individuals diagnosed using the Fukuda criteria and a subset that also fulfilled the ICC [14]. Expectedly, those fulfilling the ICC reported significantly reduced HRQoL scores compared with ME/CFS patients diagnosed using the Fukuda criteria. This current investigation uses a large sample size to provide a statistical overview of HRQoL outcomes for Australian ME/CFS patients.

SF-36 has been recommended as a sufficient measure to determine substantial reductions in HRQoL. Reeve’s empirical ME/CFS case definition recommends scores below the 25th percentile of any of the four mentioned subscales including; (i) Physical Functioning ≤ 70; (ii) Role Physical ≤ 50; (iii) Role Emotional ≤ 66.7 and (iv) Social Functioning ≤ 75 [11]. A review by Jason and colleagues reported that the SF-36 scales have adequate discrimination between ME/CFS patients and healthy individuals following Reeve’s empirical cut-off scores [15]. While Role Emotional was reported to have the worst sensitivity and specificity for ME/CFS patients, Role Physical (≤ 50), Social Functioning (≤ 62.5) and Energy/Fatigue (≤ 35) had the best sensitivity and specificity [15]. In this present investigation, few participants did not fall within these cut-offs with 11.4% obtained Social Functioning scores ≤ 62.5, 10.4% obtained Energy scores ≤ 35 and 3.5% obtained role physical scores ≤ 50.

In this investigation, low HRQoL scores were correlated with unemployment, cognition deficits in severe patients, sensory and sleep disturbances, flu-like symptoms and cardiovascular symptoms. Cognitive difficulties, pain and sleep disturbances were reported as the most common symptom amongst ME/CFS patients. We also report that physical role was significantly correlated with mental cognition, pain and gastrointestinal upsets. This investigation included a higher number of females, which has been a common observation in numerous epidemiological studies and not a limitation in this current study [16–20]. We found the females reported significantly reduced scores for physical functioning and energy/fatigue; however, scores for other domains were not significant between genders. As expected, lower scores of HRQoL were observed in ME/CFS patients who more frequently visit their general practitioner. Patient reported outcome measures in clinical practice, in particular those evaluating HRQoL, have been proposed as a means of facilitating doctor-to-patient communication, understanding patient complaints, monitoring symptoms or treatment and screening for functioning problems [21].

In this present investigation, 56.7% of ME/CFS patients were unemployed, with half receiving government issued disability payments. This is consistent with a previous investigation by Nijs and colleagues who reported a significant association between self-reported employment status and physical functioning as well as social functioning [22]. In this report, of the 54 ME/CFS patients, 50% received financial disability compensation. Low employment rates are believed to contribute to the economic burden imposed by ME/CFS [23]. Using archival data from USA, Jason and colleagues reported that the annual direct cost per ME/CFS patient was estimated between US$2,342 and US$8,675 [24]. The total costs associated with ME/CFS was approximately US$17–24 billion annually taking into consideration loss of productivity, salary variables, cost of health care providers and increased government issued disability payments.

Higher BMI had a significant impact on physical functioning, pain, general health, as well as energy/fatigue scores. The authors would like to note that physicians and patients need to carefully consider the management of physical activity to improve BMI in ME/CFS patients. Previous investigations have reported a marked onset of fatigue and poor recovery in ME/CFS patients following physical activity [1, 25]. ME/CFS patients suffer from exacerbated fatigue following physical exertion and can exercise less often and to a lesser intensity than healthy controls [22]. Few studies have examined the association of weight gain/obesity with ME/CFS. Flores and colleagues reported that overweight/obese ME/CFS patients had significantly reduced SF-36 disability outcomes [26]. Therefore, comorbid weight has consequences on physical functioning and HRQoL subsets. Impaired physical functioning and severe post-exertional malaise may contribute to inactivity as physical functioning was also associated with cardiovascular complaints.

Our findings are consistent with large studies published from the United States of America (USA), Denmark and United Kingdom [22, 27–32]. A Danish study demonstrated that HRQoL scores among those with ME/CFS were significantly reduced compared with the general population norms as well as 20 other chronic conditions including rheumatoid arthritis, numerous malignancies, angina and ischemic stroke, pulmonary disease and multiple sclerosis [32], while a study published from the United Kingdom reported significantly reduced HRQoL scores in ME/CFS patients compared to the general population in addition to other chronic conditions including depression, cancer and rheumatoid arthritis [27]. This publication also reported reduced HRQoL scores in ME/CFS patients defined using the CCC in comparison with the Fukuda criteria [27]. This is believed to be attributed to additional symptoms required to meet the CCC and ICC criteria. The Fukuda case definition is a limiting factor as symptoms are not specific, whereas the CCC and ICC definitions are highly specific for ME/CFS and encompasses more symptom categories.

The pathophysiology and aetiology of ME/CFS remain unknown resulting in diagnostic ambiguity and difficulty [33]. Without a clear understanding of the pathophysiology, no internationally recognised treatment exists, and current medical interventions focus on symptom management. A review by Bested and Marshall on the diagnosis and management of ME/CFS patients reported an estimate that only 20% of individuals with ME/CFS have been formally diagnosed and fewer have received expert medical advice [34]. There is evidence to suggest different phenotypes or profiles may explain variability between patients [35]. As this study provides evidence of the disabling effects of ME/CFS and impaired quality of life, this evidence hopes to motivate the Australian public health community. We believe this study represents the need for renewed public health policy leading to increased research outputs and thus patient care.

This publication is not without limitations: firstly, the recruitment method for this investigation was based on self-identification in response to NCNED advertisements. It is therefore unknown how many active members were potentially eligible for the study and the rate of non-responders is unable to be determined. Secondly, the survey used for this investigation is limited by self-reporting of medical history and symptoms. Third, due to the cross-sectional design of this investigation, it is also not possible to make causal inference between the examined symptoms and HRQoL domains. Prospective studies of this cohort in the future may provide further evidence towards the aetiology of ME/CFS. Fourth, the authors note that a number of HRQoL domains had lower explanatory power, however, remained statistically significant (*p* ≤ 0.001). Future research will investigate the effect of additional clinical variables, such as disease stage, symptom severity and comorbidities to gather additional information to include in our model. Fourth, data obtained during this investigation were compared to healthy population norms released 25 years ago. Finally, during symptom analysis data were categorised dichotomously as either none-mild or moderate–severe. Therefore, variation for symptom severity in ME/CFS patients may be further assessed for future investigations by categorising ME/CFS patient symptoms as either none, mild, moderate or severe.

## Conclusion

While numerous publications have reported on HRQoL outcomes for ME/CFS patients, there is limited literature using Australian patients. All ME/CFS patients included in this investigation met the Fukuda diagnostic criteria for ME/CFS and reported significantly reduced HRQoL outcomes. Lowest scores were obtained from categories including limitations due to physical role and fatigue. Low scores were associated with unemployment, mental cognition, sensory and sleep disturbances, gastrointestinal upset, cardiovascular abnormalities, changes in body temperature and flu-like symptoms. The evidence of this investigation provides confirmation of the negative effects ME/CFS has on HRQoL. Future research should examine the role of ME/CFS symptoms and demographic factors using prospective studies.

## Limitations of the study

The limitations of this investigation reported by the authors are as follows: (i) weight and height were self-reported; (ii) medical history, symptom presentation and quality of life responses were self-reported; (iii) Australian population norms used as comparative data were published 25 years ago and (iv) variations of symptom severity were not analysed with quality of life scores.

## Availability of data and material

The datasets used and/or analysed during the current study are available from the corresponding author on reasonable request.
